# Tracheobroncial colonization in coronary care unit intubated patients

**DOI:** 10.1186/1753-6561-5-S6-P67

**Published:** 2011-06-29

**Authors:** A Spyrou, J Triklis, Z Aggelpopoulou, S Tsiodras, G Saroglou

**Affiliations:** 1 Coronary Care Unit, Onassis Cardiac Surgery Center, Athens, Greece; 2 Faculty of Nursing, University of Athens, Athens, Greece; 3 Emergency Department, Onassis Cardiac Surgery Center, Athens, Greece; 4 4th Department of Medicine, University of Athens, Athens, Greece

## Introduction / objectives

Tracheobronchial tree colonization is considered an important risk factor for the development of ventilator-associated pneumonia (VAP). The aim of the present study was to examine microbiologically the respiratory tract flora of mechanically ventilated patients hospitalized in the Coronary Care Unit (CCU) of our institution for the detection of colonization with important bacterial pathogens.

## Methods

Cultures of bronchial excretions were taken the first 24h of intubation and before extubation, in a total of 39 CCU patients (mean age 68.7±11.9yrs, 79,6% men). Risk factors for colonization, including APACHE II score, Clinical Pulmonary Infection Score (CPIS), placement of an invasive device as well as the duration of mechanical ventilation (MV), were recorded.

## Results

46% of the participants with normal flora in the first 24 hours of intubation, were colonized with potential pathogens by the day of extubation. The most frequently isolated colonizers were *Acinetobacter* spp, MRSA, MSSA, *Escherichia coli*, *Candida albicans*, *Enterobacter* spp and Serratia *marcescens*. Patients who were colonized by potential pathogens had higher CPIS compared to those who were not (p <,001). Colonization with a potential pathogen was associated with placement of a nasogastric catheter (p=,048) or a central vein catheter (p=,004), with the duration of MV(p=,010) and placement of an Intra-Aortic Balloon Pump(p=,009) for a prolonged period.

## Conclusion

High rates of bacterial colonization were identified in this cohort of CCU patients. The risk factors identified will assist in the establishment of a better infection control protocol in the future.

## Disclosure of interest

None declared.

